# First description of the female of *Calamaria andersoni* Yang & Zheng, 2018 (Squamata, Calamariidae), with an expanded diagnosis

**DOI:** 10.3897/BDJ.13.e165597

**Published:** 2025-12-15

**Authors:** Tierui Zhang, Yuhao Xu, Nguyen Van Tan, Nikolay Poyarkov, Lifang Peng, Jundong Deng, Xinge Wang, Song Huang

**Affiliations:** 1 Anhui Province Key Laboratory of the Conservation and Exploitation of Biological Resource, College of Life Sciences, Anhui Normal University, Wuhu, China Anhui Province Key Laboratory of the Conservation and Exploitation of Biological Resource, College of Life Sciences, Anhui Normal University Wuhu China; 2 State Key Laboratory of Plateau Ecology and Agriculture, Qinghai University, Xining, China State Key Laboratory of Plateau Ecology and Agriculture, Qinghai University Xining China; 3 The School of Medicine & Pharmacy, Duy Tan University, Da Nang, Vietnam The School of Medicine & Pharmacy, Duy Tan University Da Nang Vietnam; 4 Center for Entomology & Parasitology Research, Da Nang, Vietnam Center for Entomology & Parasitology Research Da Nang Vietnam; 5 Department of Vertebrate Zoology, Lomonosov Moscow State University, Moscow, Russia Department of Vertebrate Zoology, Lomonosov Moscow State University Moscow Russia

**Keywords:** Reed Snake, morphology, phylogenetics, sexual dimorphism, China

## Abstract

**Background:**

*Calamaria andersoni* Yang & Zheng, 2018, was originally described from a single adult male collected in Dehong Prefecture, Yunnan Province, China. Until now, no female specimens had been reported, limiting understanding of sexual dimorphism and diagnostic variation. Based on newly collected material from Mangshi City and Lianghe County, we identified five males and one female as *C. andersoni* through morphological comparisons and mitochondrial cytochrome b gene analysis.

**New information:**

We provide the first morphological description of the female and revise the species diagnosis based on both sexes. The female is distinguishable by a higher number of ventrals and fewer subcaudals. Genetic data confirmed all specimens as belonging to a well-supported clade of *C. andersoni*, which is 9.8–10.1% divergent from *C. yunnanensis*. The known distribution of the species is extended to lower elevations (1,185–1,315 m a.s.l.) within tropical evergreen forests of Dehong. Given its relatively broad elevational range and occurrence in mature forests but no apparent population threats, we propose its conservation status as Least Concern (LC) under IUCN criteria. These findings provide new insight into sexual dimorphism, distribution, and species-level boundaries in *Calamaria*, emphasising the importance of continued surveys in underexplored montane regions of Yunnan and adjacent northern Myanmar.

## Introduction

The Southeast Asian reed snake genus *Calamaria* H. Boie in Boie (1827), comprises small, burrowing, forest-dwelling, nonvenomous snakes. The genus currently includes 70 species distributed from eastern China and the Ryukyu Islands in the north, through Northeastern India, Vietnam, Laos, Cambodia, Thailand, and the Malay Peninsula to Myanmar in the west, and southward to Sulawesi, Seram, and the Philippines (Poyarkov et al. 2023, Uetz et al. 2025). In recent years, extensive field sampling combined with molecular phylogenetic analyses has led to the discovery and description of more than ten new species in this genus (e.g., Yang and Zheng 2018, Poyarkov et al. 2019, Lee 2021, Yeung et al. 2022, Cai et al. 2023). To date, seven species of *Calamaria* have been recorded from China: *C. arcana* Yeung, Lau & Yang; *C. andersoni* Yang & Zheng; *C. berezowskii* Günther; *C. jinggangensis* Cai, Jiang, Wu, Huang, Fei & Ding; *C. pavimentata* Duméril, Bibron & Duméril; *C. septentrionalis* Boulenger; and *C. yunnanensis* Chernov (Cai et al. 2023, Liang et al. 2024, Uetz et al. 2025).

The Anderson’s Reed Snake, *Calamaria andersoni*, was described based on a single adult male specimen collected from Tongbiguan Township, Yingjiang County, Dehong Dai and Jingpo Autonomous Prefecture, Yunnan Province, China (Yang and Zheng 2018). According to Yang and Zheng (2018), a mitochondrial Cyt *b* gene analysis revealed that *C. andersoni* is most closely related to *C. yunnanensis*, yet it can be distinguished from the latter by several morphological characters: presence of a preocular scale (vs. absent in *C. yunnanensis*), fewer ventral scales in males (171 vs. 179–201), a higher number of subcaudals in males (23 vs. 15–22), all dorsal scale rows pigmented (vs. outermost one or two dorsal scale rows unpigmented), and ventral scales with dark lateral corners (vs. immaculate ventrals in *C. yunnanensis*). However, no comparisons have yet been made between females of these two species. Despite its initial discovery, *C. andersoni* has remained poorly known due to the lack of additional specimens, particularly females. This gap has limited our understanding of the species' diagnostic traits and distribution.

**Figure 1.**
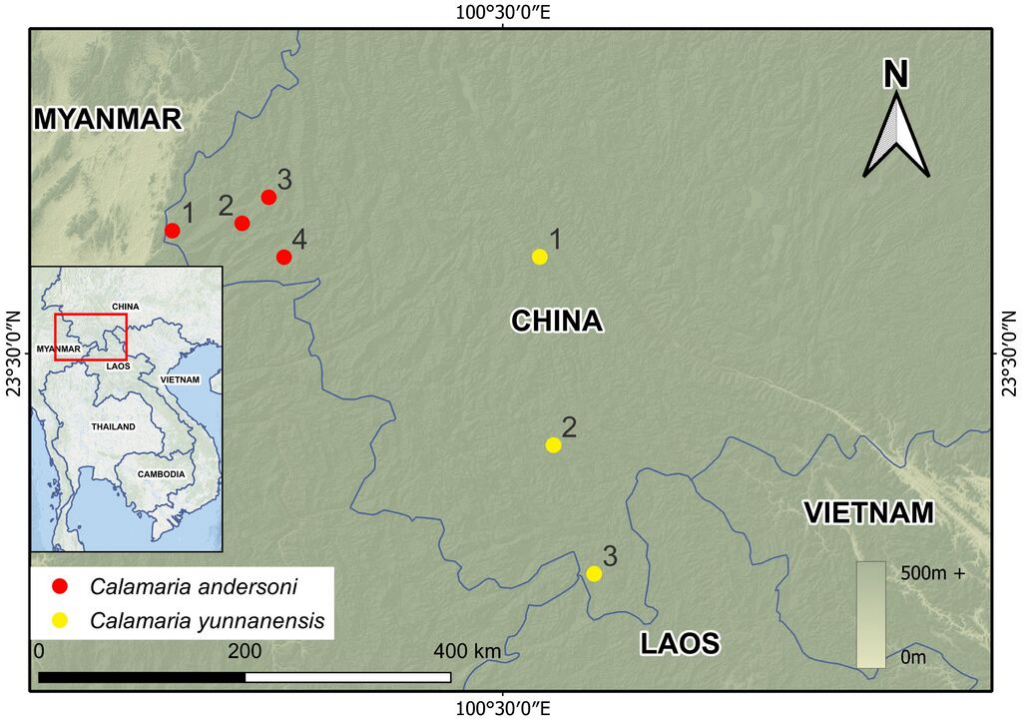
Distribution ranges of *Calamaria andersoni* and *C. yunnanensis* in Yunnan, China. Numbers correspond to locality records (see Table 1 for locality details). Map was created in QGIS using open-source DEM data (NASA JPL 2013).

During a herpetofaunal survey in Yunnan Province, China, six specimens of *Calamaria* (one female and five males) were collected from Mangshi City and Lianghe County in Dehong Dai and Jingpo Autonomous Prefecture (Fig. 1, Table 1). Morphological comparisons and molecular phylogenetic analyses confirmed their identity as *Calamaria andersoni*. In this study, we provide the first morphological description of the female *C. andersoni*, update the known distribution of the species, and revise its diagnostic characters.

## Materials and methods

### Sampling

Five adult male specimens and one adult female specimen of *Calamaria* were collected from Dehong Dai and Jingpo Autonomous Prefecture, Yunnan Province, China (Fig. 1). The specimens were humanely euthanised using a 0.7% solution of tricaine methanesulfonate (MS-222) through intraperitoneal injection. Fresh liver tissue was extracted and immediately preserved in 95% ethanol for molecular analyses. The specimens were fixed and stored in 75% ethanol for long-term preservation and are deposited in the museum collections of Anhui Normal University (ANU), Anhui, China, and Qinghai University (QHU), Qinghai, China. All procedures complied with the Wildlife Protection Law of China and were approved by the Institutional Ethics Committee of Qinghai University (protocol number PJ202501-89) and the Animal Ethics Committee of Anhui Normal University (protocol number AHNU-ET2024169).

### Molecular phylogeny

Total genomic DNA was extracted from preserved liver tissue with OMEGA Tissue DNA Kit D3396 (Omega Bio-Tek, Norcross, GA, USA). A fragment of the mitochondrial cytochrome *b* (Cyt *b*) gene was amplified using the primer pair L14910 (5’-GACCTGTGATMTGAAACCAYCGTTGT-3’) and H16064 (5’-CTTTGGTTTACAAGAACAATGCTTTA-3’) (Burbrink et al. 2000). The double-stranded products were sequenced by General Biosystems (Anhui) Corp. Ltd (Chuzhou, China), and raw sequences were assembled using SeqMan in the DNASTAR software package (Burland 2000).

A total 44 sequences from 14 known *Calamaria* species and three out-group species, *Elaphe quatuorlineata* (Lacépède), *Orientocoluber spinalis* (Peters), and *Lycodon rufozonatus* Cantor, were obtained from GenBank or newly sequenced and incorporated into our dataset (Suppl. material 1 Table S1). DNA sequences were aligned by the Clustal W algorithm with default parameters (Thompson 1997) and trimmed with gaps partially deleted in MEGA X (Kumar et al. 2018). Bayesian inferences (BI) were conducted in MRBAYES v. 3.2.7a (Ronquist et al. 2012) under the GTR + I + G model on Phylosuite v1.2.3 (Zhang et al. 2020, Xiang et al. 2023). In the BI analysis, three independent runs were conducted with 1 × 10^7^ generations and sampled every 1,000 generations, with the first 25% of samples were discarded as burn-in. In the ML analysis, the bootstrap consensus tree was inferred from 1000 replicates. Maximum likelihood (ML) was conducted under the best-fit substitution model (GTR + I + G) in RaxmlGUI 1.3 (Silvestro and Michalak 2011). Bootstrap proportions (BSP) were investigated with 1,000 bootstrap replicates using the fast-bootstrapping algorithm. Uncorrected pairwise genetic distances (*p*-distance) of Cyt *b* gene among congeners were calculated with MEGA X (Kumar et al. 2018).

### Morphological comparison

Terminology and measurements follow Inger and Marx (1965). Measurements were taken with a digital slide caliper to the nearest 0.1 mm, except for body and tail lengths, which were measured to the nearest millimetre with a measuring tape. The number of ventral scales was counted according to Dowling (1951). The numbers of dorsal scale rows are given at one head length behind the head, at midbody, and at one head length before the vent. The sex was determined by dissection (inspection of gonads). Maxillary teeth of the specimens were counted by examining both maxillae using a dissecting pin under a binocular microscope prior to preservation.

The following measurements (all in mm) and counts were taken: head length (HL, from snout tip to jaw angles); head width (HW, measured at the widest point of the head); eye diameter (ED, horizontal); eye-mouth distance (Eye-MouthD, measured from the anterior point of the eye to the mouth gap); naris–eye distance, measured from the anterior point of the eye to the medial point of the nostril (NarEye); interorbital distance, the straight-line distance between the eyes at the border of the supraoculars (IOD); snout-vent length (SVL); tail length (TaL); total length (TL); ratio of tail length/total length (TaL/TL); number of maxillary teeth (MT); dorsal scale rows number (DSR); supralabial scales (SL); number of supralabials touching the eye (SL-E); infralabial scales (IL); preocular scales (PrO); postocular scales (PoO); subcaudal scales (SC); ventral scales (VEN); contact of posterior chin shields (CPCS). Asymmetric characters are given in left/right order. Other abbreviations. Mt. = Mountain; NP = National Park; a.s.l. = above sea level. Museums, private collections and biorepository abbreviations are shown in Suppl. material 1 Table S2.

## Taxon treatments

### Calamaria andersoni

Yang & Zheng, 2018

B0E981BD-0A3A-596B-9C98-C51243F78E09

#### Materials

- **Type status:** Other material. **Occurrence:** catalogNumber: ANU ZR25022; individualCount: 1; sex: male; lifeStage: adult; occurrenceID: E82D8737-6380-5668-BC4A-0939E497D4F9; **Taxon:** taxonID: urn:lsid:biosci.ohio-state.edu:osuc_names:275502; scientificNameID: *Calamaria andersoni*; scientificName: *Calamaria andersoni*; class: Reptilia; order: Squamata; family: Calamariidae; genus: Calamaria; specificEpithet: *andersoni*; scientificNameAuthorship: Yang & Zheng, 2018; **Location:** country: China; countryCode: CN; stateProvince: Yunnan; locality: Mangshi City, Dehong Dai and Jingpo Autonomous Prefecture; verbatimElevation: 1315 m; verbatimLatitude: 24.520531°N; verbatimLongitude: 98.589152°E; verbatimCoordinateSystem: WGS84; **Event:** eventDate: 5/3/2025; eventRemarks: collected by J.D. Deng; **Record Level:** language: en; collectionCode: Reptilia; basisOfRecord: PreservedSpecimen
- **Type status:** Other material. **Occurrence:** catalogNumber: HS R21036; individualCount: 1; sex: female; lifeStage: adult; occurrenceID: C3122350-A780-5D0A-A867-21B1B0CFB089; **Taxon:** taxonID: urn:lsid:biosci.ohio-state.edu:osuc_names:275502; scientificNameID: *Calamaria andersoni*; scientificName: *Calamaria andersoni*; class: Reptilia; order: Squamata; family: Calamariidae; genus: Calamaria; specificEpithet: *andersoni*; scientificNameAuthorship: Yang & Zheng, 2018; **Location:** country: China; countryCode: CN; stateProvince: Yunnan; locality: Lianghe County, Mangdong Town, Dehong Dai and Jingpo Autonomous Prefecture; verbatimElevation: 1185 m; verbatimLatitude: 24.674581°N; verbatimLongitude: 98.221510°E; verbatimCoordinateSystem: WGS84; **Event:** eventDate: 6/15/2021; eventRemarks: collected by J.D. Deng; **Record Level:** language: en; collectionCode: Reptilia; basisOfRecord: PreservedSpecimen
- **Type status:** Other material. **Occurrence:** catalogNumber: ANU ZR24017; individualCount: 1; sex: male; lifeStage: adult; occurrenceID: AF0FF5F1-E3D6-5819-9139-1B313EE7FD69; **Taxon:** taxonID: urn:lsid:biosci.ohio-state.edu:osuc_names:275502; scientificNameID: *Calamaria andersoni*; scientificName: *Calamaria andersoni*; class: Reptilia; order: Squamata; family: Calamariidae; genus: Calamaria; specificEpithet: andersoni; scientificNameAuthorship: Yang & Zheng, 2018; **Location:** country: China; countryCode: CN; stateProvince: Yunnan; locality: Lianghe County, Mangdong Town, Dehong Dai and Jingpo Autonomous Prefecture; verbatimElevation: 1185 m; verbatimLatitude: 24.674581°N; verbatimLongitude: 98.221510°E; verbatimCoordinateSystem: WGS84; **Event:** eventDate: 7/8/2024; eventRemarks: collected by J.D. Deng; **Record Level:** language: en; collectionCode: Reptilia; basisOfRecord: PreservedSpecimen
- **Type status:** Other material. **Occurrence:** catalogNumber: QHU R2025016; individualCount: 1; sex: male; lifeStage: adult; occurrenceID: 4971066E-766B-546C-81B9-ED2A161A67BD; **Taxon:** taxonID: urn:lsid:biosci.ohio-state.edu:osuc_names:275502; scientificNameID: *Calamaria andersoni*; scientificName: *Calamaria andersoni*; class: Reptilia; order: Squamata; family: Calamariidae; genus: Calamaria; specificEpithet: *andersoni*; scientificNameAuthorship: Yang & Zheng, 2018; **Location:** country: China; countryCode: CN; stateProvince: Yunnan; locality: Lianghe County, Mangdong Town, Dehong Dai and Jingpo Autonomous Prefecture; verbatimElevation: 1185 m; verbatimLatitude: 24.674581°N; verbatimLongitude: 98.221510°E; verbatimCoordinateSystem: WGS84; **Event:** eventDate: 5/5/2025; eventRemarks: collected by J.D. Deng; **Record Level:** language: en; collectionCode: Reptilia; basisOfRecord: PreservedSpecimen
- **Type status:** Other material. **Occurrence:** catalogNumber: QHU R2025017; individualCount: 1; sex: male; lifeStage: adult; occurrenceID: 253E96DA-1AC2-5A2E-914F-D2CCFB7A5814; **Taxon:** scientificNameID: *Calamaria andersoni*; scientificName: *Calamaria andersoni*; class: Reptilia; order: Squamata; family: Calamariidae; genus: Calamaria; specificEpithet: *andersoni*; scientificNameAuthorship: Yang & Zheng, 2018; **Location:** country: China; countryCode: CN; stateProvince: Yunnan; locality: Lianghe County, Mangdong Town, Dehong Dai and Jingpo Autonomous Prefecture; verbatimElevation: 1185 m; verbatimLatitude: 24.674581°N; verbatimLongitude: 98.221510°E; verbatimCoordinateSystem: WGS84; **Event:** eventDate: 5/5/2025; eventRemarks: collected by J.D. Deng; **Record Level:** language: en; collectionCode: Reptilia; basisOfRecord: PreservedSpecimen
- **Type status:** Other material. **Occurrence:** catalogNumber: QHU R2025018; individualCount: 1; sex: male; lifeStage: adult; occurrenceID: 8E047098-C3BE-5A2B-8F70-C58A4F2436B6; **Taxon:** scientificNameID: *Calamaria andersoni*; scientificName: *Calamaria andersoni*; class: Reptilia; order: Squamata; family: Calamariidae; genus: Calamaria; specificEpithet: andersoni; scientificNameAuthorship: Yang & Zheng, 2018; **Location:** country: China; countryCode: CN; stateProvince: Yunnan; locality: Lianghe County, Mangdong Town, Dehong Dai and Jingpo Autonomous Prefecture; verbatimElevation: 1185 m; verbatimLatitude: 24.674581°N; verbatimLongitude: 98.221510°E; verbatimCoordinateSystem: WGS84; **Event:** eventDate: 5/5/2025; eventRemarks: collected by J.D. Deng; **Record Level:** language: en; collectionCode: Reptilia; basisOfRecord: PreservedSpecimen

#### Description of the female specimen (HS R21036)

**Figure 2.**
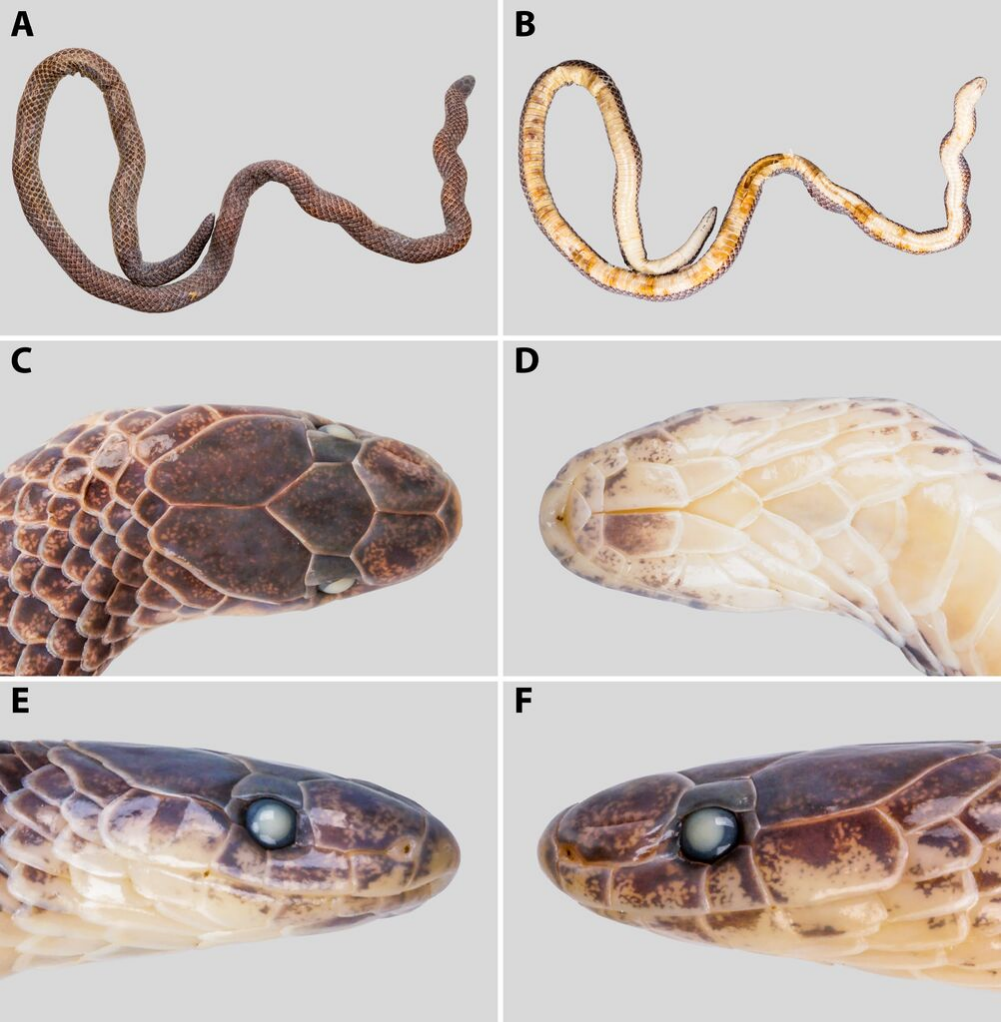
*Calamaria andersoni* in preservative. Specimen HS R21036, adult female: **A** general dorsal view; **B** general ventral view; **C** dorsal view of head; **D** ventral view of head; **E** right lateral view of head; **F** left lateral view of head. Photographs by T.R. Zhang

**Measurements and scalation** (Fig. 2). Specimen HS R21036 is in excellent condition. Body slender and cylindrical (SVL 294 mm, TL 312 mm). Tail short, not as thick as body (TaL 18 mm, TaL/TL 5.8%); tail uniformly cylindrical in the anterior part, tail slowly tapering anteriorly, then abruptly tapering at tip; head small, elliptical in dorsal view (HL 7.8 mm, HW 4.2 mm, HH 3.5 mm); eye small and round (ED 0.9 mm), larger than eye-mouth distance (Eye-MouthD 0.9 mm), ED/HL 11.5%.

Rostral wider than high, portion visible from above almost equal to the length of the prefrontal suture; prefrontal slightly shorter than frontal, not entering orbit, in contact with 1^st^ and 2^nd^ supralabial; frontal hexagonal, longer than wide, about 3.0 times the maximum width of the supraocular; paraparietal surrounded by six scales; preocular 1/1, higher than wide, slightly higher than postocular, not as high as eye diameter; postocular 1/1, higher than wide; nasals small, barely surrounding nostrils, surrounded by the 1^st^ supralabial, rostral, and prefrontal; supralabials 4/4, 2^nd^ and 3^rd^ entering orbit, 4^th^ largest, relative supralabial width 4>2>1>3; mental triangular, not in contact with the anterior chin shields; infralabials 5/5, first three pairs touching anterior chin shields, first pair meeting in midline, 4^th^ largest; anterior chin shields longer than wide, pentagonal, meeting in midline; posterior chin shields shorter than the anterior ones, touching at their foremost ends and separated posteriorly by first gular scales.

Dorsal scales in 13 rows throughout the body, reducing to six rows above the fifth subcaudal and to four rows above the second to last subcaudals. Dorsal scales homogeneous in size, entirely smooth; vertebral row not enlarged. Ventrals 186. Anal plate undivided. Subcaudals 14, paired and smooth; terminal scale single and rigid.

**Dentition**. Maxillary teeth enlarged, uniformly arranged, nine on each side (9/9).

**Colouration in preservative** (Fig. 2). Dorsal surface of the head, body, and tail light to dark brown, with a slightly faded pattern compared to the colouration in life. Lateral stripes are obscure or absent. The dorsal head scales are uniformly brown, with faint lighter mottling along the edges of the supralabials. The eye appears cloudy white due to fixation. The ventral surface is pale cream to yellowish white, with irregular brown to dark brown mottling along the outer margins of the ventral scales, especially pronounced on the anterior half of the body. The posterior venter and subcaudals are more uniformly cream-coloured with sparse dark markings. The chin and throat are pale, with scattered brown speckling on the infralabials and gular region. The tail tip is slightly darker dorsally and lighter ventrally, and has the same color as the adjacent trunk areas.

#### Description of additional specimens

**Morphology.** Body slender and cylindrical [the longest known specimen is 312 mm long (HS R21036); the longest known male is 295 mm long (SVL 269 mm, TaL 26 mm; ANU ZR24017); tail uniformly cylindrical in the anterior part, tail slowly tapering anteriorly, then abruptly tapering at tip (TaL/TL 8.8–9.2% in males (n=6) and 5.8% in females (n=1)).

**Body scalation.** Dorsal scales in 13 rows throughout the body, reducing to 6 rows above the 4^th^–9^th^ subcaudals and to 4 rows above the second and 5^th^ to last subcaudals. Dorsal scales homogeneous in size, entirely smooth; vertebral row not enlarged. Ventrals 164–172 in males (n = 6), 186 in the female (n = 1). Anal plate undivided. Subcaudals 20–23 in males (n = 6), 14 in the female (n = 1), paired and smooth; terminal scale single and rigid.

**Head scalation.** Rostral wider than high; portion visible from above almost equal to the length of the prefrontal suture; prefrontal slightly shorter than frontal, not entering orbit, in contact with 1^st^ and 2^nd^ supralabial; frontal hexagonal, longer than wide; paraparietal surrounded by six scales; preocular 1/1, higher than wide, slightly higher than postocular, not as high as eye diameter; postocular 1/1, higher than wide; nasals small, barely surrounding nostrils, surrounded by 1^st^ supralabial, rostral and prefrontal; supralabials 4/4, 2^nd^ and 3^rd^ entering orbit, 4^th^ largest, relative supralabial width 4>2>1>3; mental triangular, not in contact with the anterior chin shields; infralabials 5/5, first three pairs touching anterior chin shields, first pair meeting in midline, 4^th^ largest; anterior chin shields longer than wide, pentagonal, meeting in midline; posterior chin shields shorter than the anterior ones, touching anteriorly and separated posteriorly by first gular scales.

**Dentition.** Maxillary teeth enlarged, uniformly arranged, nine on each side (9/9).

**Figure 3.**
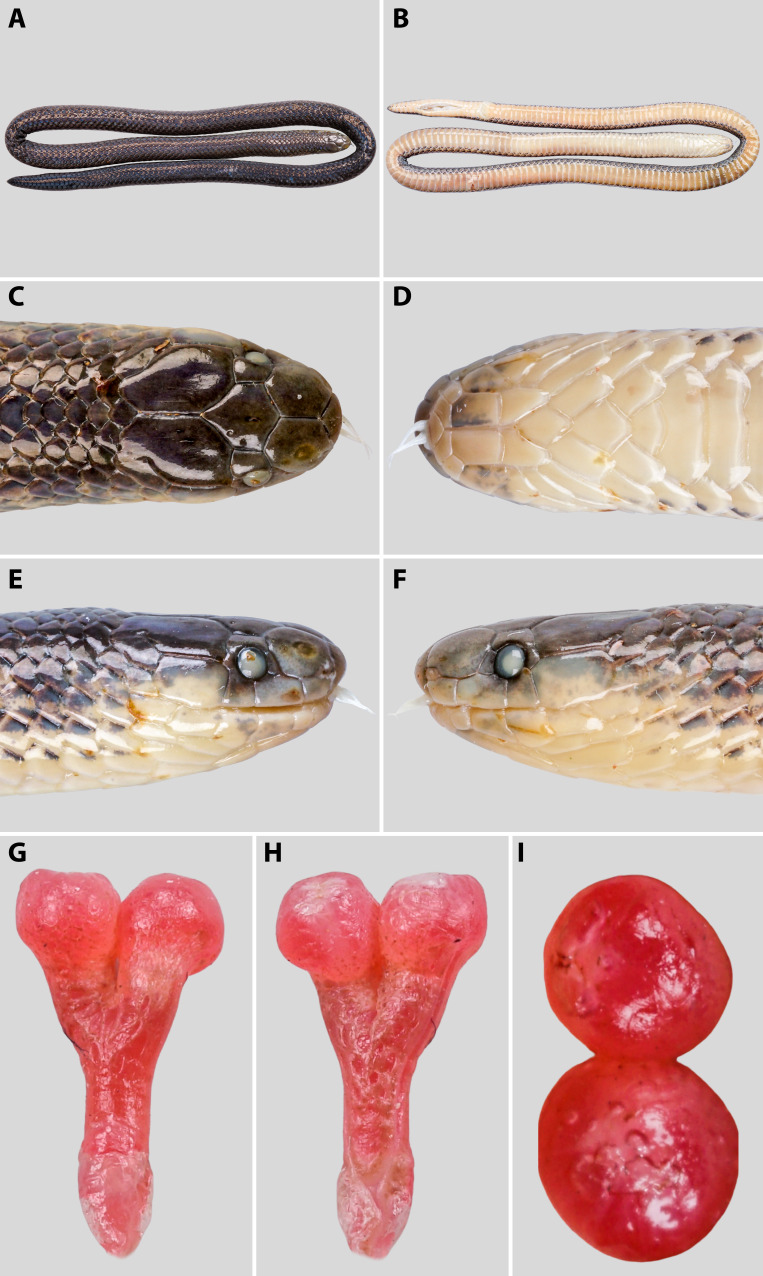
*Calamaria andersoni* in preservative. Specimen QHU 2025018, adult male. **A** general dorsal view; **B** general ventral view; **C** dorsal view of head; **D** ventral view of head; **E** right lateral view of head; **F** left lateral view of head; **G** sulcal view of everted hemipenes; **H** lateral view of hemipenes; **I** apical view of hemipenial lobes. Photographs by T.R. Zhang.

**Hemipenis** (based on fully everted organ of adult male specimen QHU 2025018; Fig. 3 G-I). The hemipenis is bilobed, relatively short and thick, with two hemispherical lobes approximately equal in size. Lobes moderately expanded and rounded distally. The organ is deeply bifurcated, with the bifurcation starting at approximately the level of the 4^th^ subcaudal scale and extending terminally to the tip of each lobe. The entire organ extends posteriorly to the level of the 7^th^ subcaudal scale. Sulcus spermaticus is single, centrally positioned, bifurcating shortly before the base of the lobes, and terminating apically on each lobe. The truncus (stem) is cylindrical, unornamented, and smooth, lacking spines, folds, or calyces. The surface of both lobes and the proximal part of the truncus is smooth throughout, without papillate or spinulate structures. In preservative, the organ is uniformly pinkish to pale reddish, with the lobes slightly more pigmented than the basal stem.

**Figure 4.**
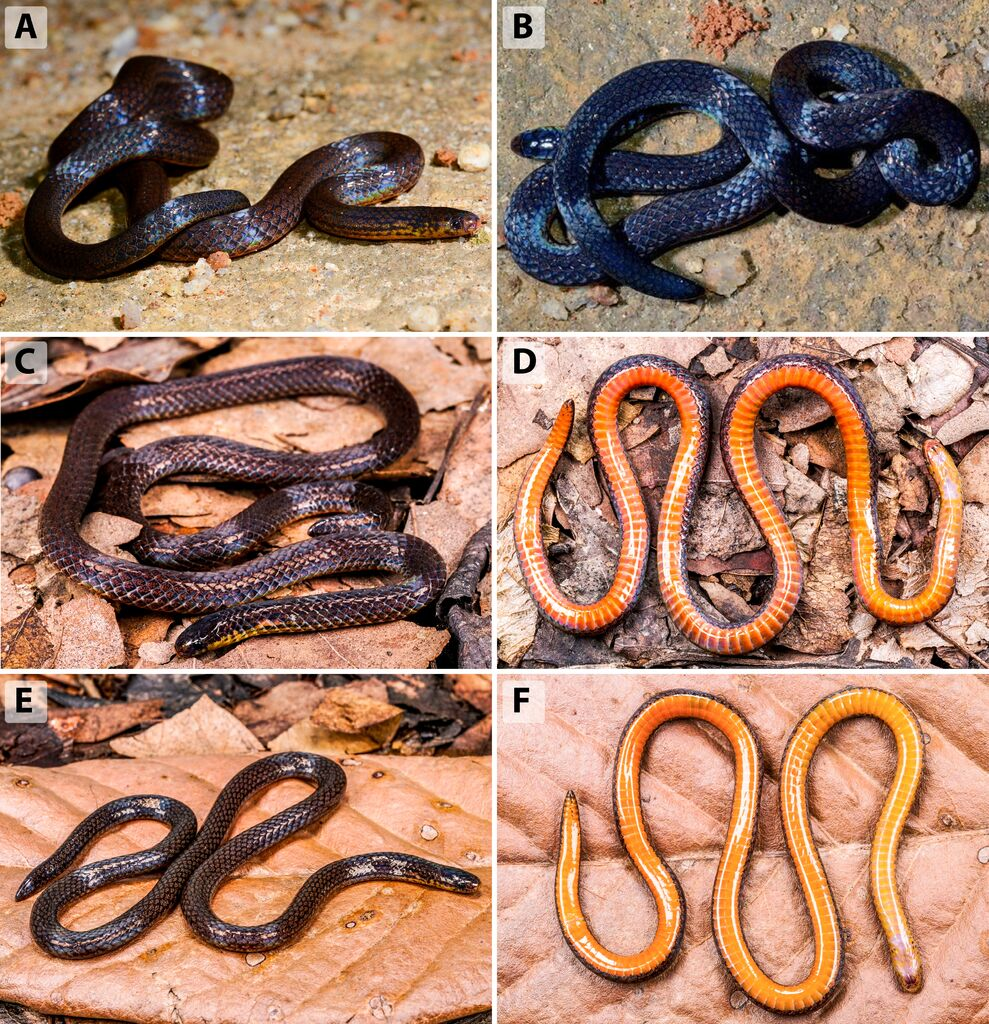
*Calamaria andersoni* in life from Yunnan, China: specimen SYS r001699 (adult male, holotype) from Tongbiguan, Yingjiang, Dehong (A-B); specimen ANU ZR25022 (adult male) from Mangshi, Dehong (C-D); specimen ANU ZR24017 (adult male) from Mangdong, Lianghe, Dehong (E-F). Photographs by J.H. Yang (A-B); T.R. Zhang (C-F).

**Colouration in life** (Fig. 4 A-F). Dorsal surface of head, body, and tail uniformly dark brown to blackish, with subtle iridescence visible under direct light. A series of indistinct narrow dark stripes is present on the lateral sides of the body, often only faintly visible. Dorsal scales lack any conspicuous markings or collars. Supralabials and lower jaw are dark brown, with diffuse yellow to orange suffusion on the infralabials and throat region. Ventral surface bright orange to orange-yellow, bordered laterally by a narrow line of dark pigmentation at the outermost edges of the ventral scales. No light-coloured rings or collar markings are present on the nape or tail. The colouration of the tail tip is similar with that of the dorsum or slightly paler ventrally.

**Variation.** Species variation is discussed based on the holotype (SYS r001699; described in Yang and Zheng 2018) and six additional specimens reported in this study (see Table 2, Figs 2, 3 A-F, the overview measurements are listed in Suppl. material 1 Table S4).

#### Revised diagnosis.

*Calamaria andersoni* can be distinguished from its congeners by the following combination of characters: (1) nine enlarged maxillary teeth; (2) body wider than high; prefrontal shorter than frontal, contacting the first two supralabials; (3) mental not in contact with the anterior chin shields; (4) single preocular and postocular; (5) four supralabials, with the 2^nd^ and 3^rd^ contacting the eye; (6) five infralabials; five scales surrounding the paraparietal; (7) ventrals 164–172 in males, 186 in the female; subcaudals 20–23 in males, 14 in the female, paired; tail relatively short (8.8–9.2% of total length in males, 5.8% in the female), tapering gradually anteriorly and abruptly at the tip; (8) dorsal surface of the head, body, and tail uniformly dark brown to blackish; (9) no light-coloured rings or blotches on the neck or tail; (10) ventral surface bright orange to orange-yellow, bordered laterally by a narrow line of dark pigmentation along the outermost edges of the ventral scales.

#### Natural history, distribution, and conservation status.

*Calamaria andersoni* is currently known only from tropical evergreen forests in Dehong Dai and Jingpo Autonomous Prefecture, Yunnan Province, China, specifically from Tongbiguan (Yingjiang County), Mangshi City, and Mangdong Town (Lianghe County), at elevations around 1,520 m a.s.l. The species has been observed at night in moist, mossy microhabitats within mature forest environments. This region harbours high herpetofaunal diversity, and *C. andersoni* occurs sympatrically with several other snake species, including *Trimeresurus popeiorum* Smith, *Ovophis jenkinsi* Qiu, Wang, Xia, Jiang, Zeng, Wang, Li & Shi, *Lycodon fasciatus* (Anderson), and Oligodon cf. albocinctus (Cantor) (personal observations during field survey in May, 2025). As the type locality lies near the international border, it is plausible that the species also occurs in adjacent mountainous regions of northern Myanmar (Kachin State) (Fig. 1). Given the limited available information on its ecology, population size, and potential threats, we recommend that *Calamaria andersoni* be classified as Least Concern (LC) under the IUCN Red List Categories and Criteria (IUCN 2024).

## Analysis

**Figure 5.**
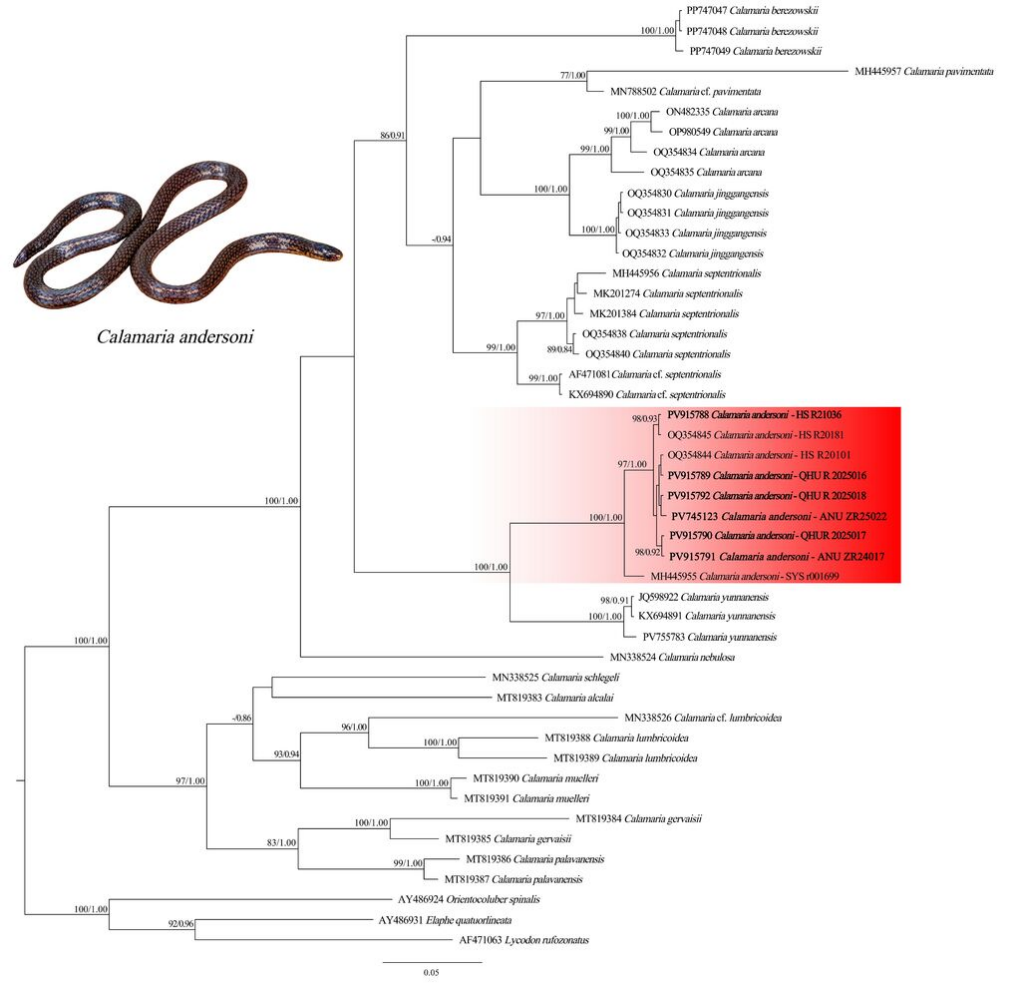
ML-dendrogram of *Calamaria* species based on the analyses of 1,105 base pairs of the mitochondrial Cyt *b* gene. Values at nodes show Bootstrap Support values (BSP) and Bayesian posterior probabilities (BPP), respectively. Values below 0.70 (BPP) or 75 (BSP) are shown as “–” or omitted.

In this study, 1,060 base pairs (bp) of Cyt *b* sequences were successfully included in the final alignment, including six newly generated sequences of *Calamaria andersoni*. Both ML and BI analyses yielded highly congruent topologies (Fig. 5), consistent with findings from previous studies (Cai et al. 2023, Liang et al. 2024).

The genus *Calamaria* was recovered as a monophyletic group within the scope of the current sampling (BSP = 100, BPP = 1.00). All *Calamaria andersoni* individuals clustered within a distinct and well-supported clade (BSP = 100, BPP = 1.00), forming a sister species to *C. yunnanensis*. Infraspecific uncorrected *p*-distances among the *C. andersoni* specimens were 0.0%–2.5% (Suppl. material 1 Table S1). Interspecific *p*-distances between *C. andersoni* and other analysed *Calamaria* species ranged from 9.8%–10.1% (versus *C. yunnanensis*) to 21.7%–21.8% (versus *C. alcalai* Weinell, Leviton & Brown), consistent with levels of genetic divergence observed among recognized species in the genus.

## Discussion

This study significantly expands our knowledge of *Calamaria andersoni*, a species previously known only from the male holotype. The newly collected material includes the first adult female specimen, allowing for the first description of sexual dimorphism in this taxon: (1) number of ventrals: 186 in female (n=1) vs. 164–172 in males (n=6); (2)number of subcaudals: 14 in female vs. 20–23 in males; (3) TaL/TL ratio: 5.8% in female vs. 8.0%–9.1% in males. These differences align with general patterns of sexual dimorphism reported across the genus Calamaria (Inger and Marx 1965, Poyarkov et al. 2019, Yeung et al. 2022). However, examination of additional female specimens are still needed to confirm these traits.

**Figure 6.**
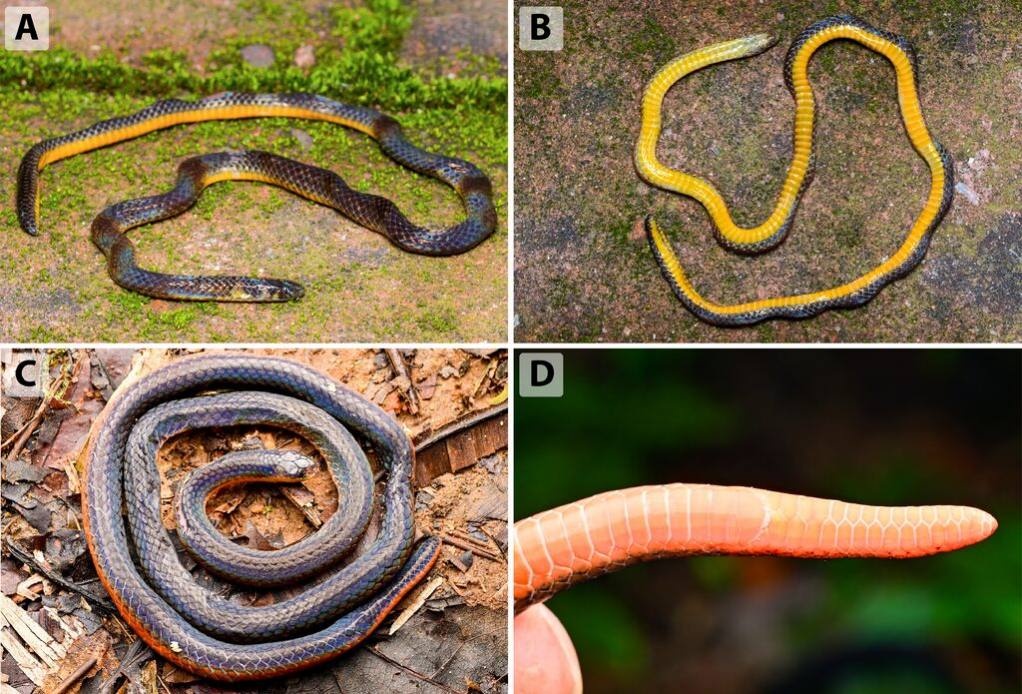
*Calamaria yunnanensis* in life from Yunnan, China: specimen QHU R2024054 (adult male) from Mt. Wanzhang, Simao (A-B); specimen QHU R2024055 (adult female) from Mengsong, Mengla, Xishuangbanna (C-D). Photographs by T.Y. Zhang (A-B), and F. Gao (C-D).

The revised diagnosis of *C. andersoni*, based on both sexes, clarifies its distinction from closely related species, particularly *C. yunnanensis*. For the latter, we follow the revised taxonomic concept proposed by Lee (2021). In addition, we examined two additional specimens of *C. yunnanensis* from Yunnan Province (see Table 1, Fig. 6A–D). *Calamaria andersoni* differs from *C. yunnanensis* in the following characters: (1) presence of a preocular scale (vs. absent); (2) smaller maximum snout-vent length in females (294 mm vs. 490 mm); (3) fewer ventral scales in males (maximum 172 vs. more than 184); (4) lower number of subcaudals in females (14 vs. 16–19); (5) uniformly pigmented dorsal scale rows (vs. outermost one or two rows unpigmented); (6) ventral scales with dark lateral corners (vs. immaculate ventrals); (6) the posterior chin shields contact at their foremost ends (vs. at anterior 1/2).

Furthermore, our new records significantly expand the known distribution of *Calamaria andersoni*, extending its range within Dehong Prefecture to include Mangshi City and Lianghe County. These localities lie at lower elevations (1,185–1,315 m a.s.l.) compared to the type locality at 1,520 m indicating that the species may occupy a broader elevational gradient within tropical evergreen forests than previously known.

Finally, given that we still know little about the natural history of *Calamaria andersoni*, targeted field surveys and ecological research are urgently needed to assess population size, reproductive biology, and the conservation status of this poorly known species.

## Supplementary Material

XML Treatment for Calamaria andersoni

92C283BA-E4CE-5086-B6E0-12F9F6ED196F 10.3897/BDJ.13.e165597.suppl1 Supplementary material 1 Tables S1–S4 Data type occurences and morphological Brief description Table S1. DNA sequences, voucher specimens, and GenBank accession numbers of the genus *Calamaria* and outgroup taxa used in this study.
Table S2. Museums, private collections and biorepository abbreviations mentioned in this study.
Table S3. Uncorrected *p*-distances (%) among *Calamaria* species based on 1,105 base pairs from the mitochondrial genes Cyt *b.* The serial numbers in Table S3 are consistent with those in Table S1.
Table S4. Overview measurements of the examined specimens of *Calamaria andersoni* and *C. yunnanensis* from Yunnan, China. File: oo_1467541.doc https://binary.pensoft.net/file/1467541 Tierui Zhang, Yuhao Xu, Tan Van Nguyen, Nikolay A. Poyarkov, Lifang Peng, Jundong Deng, Xinge Wang, Song Huang

## Figures and Tables

**Table 1.** List of localities of the *Calamaria andersoni* and *C. yunnanensis* appearing on Fig. 1. **Symbols**: (1) = Number on the map; (2) = Verified by morphology data (yes/no); (3) = Verified by molecular data (yes/no). Data is derived from own work (this study) and the literature (column 5), Cai et al. (2023) provides only molecular data.

Number on the map	Verified by morphology data	Verified by molecular data	Location	Sources
***Calamaria andersoni***				
1	yes	yes	Tongbiguan, Yingjiang, Dehong, Yunnan, China (type locality)	Yang and Zheng (2018)
2	yes	yes	Mangdong, Lianghe, Dehong, Yunnan, China	this study
3	no	yes	Tengchong, Baoshan, Yunnan, China	Cai et al. (2023)
4	yes	yes	Mangshi, Dehong, Yunnan, China	Cai et al. (2023); this study
***Calamaria yunnanensis***				
1	yes	no	Jingdong, Pu’er, Yunnan, China (type locality)	Lee (2021)
2	yes	yes	Mt. Wanzhangshan, Simao, Yunnan, China	Lee (2021); this study
3	yes	no	Mengsong, Mengla, Xishuangbanna, Yunnan, China	this study

**Table 2.** Main morphometric and meristic characters of the examined specimens of *Calamaria andersoni* and *C. yunnanensis* from Yunnan, China. Notes: n/a = not available; * = holotype; M = male; F = female; SM = subadult male; SVL = snout-vent length; TaL = tail length; TaL/TL = ratio of tail length/total length; MT = number of maxillary teeth; DSR = dorsal scale rows number; VEN = ventral scales; SC = subcaudal scales; SL = supralabial scales; SL-E = number of supralabials touching the eye; PrO = preocular scales; PoO = postocular scales; IL = infralabial scales; CPSC = contact of posterior chin shields.

Specimen Number	Sex	Locality	SVL (mm)	TaL (mm)	TaL/TL(%)	MT	DSR	VEN	SC	SL	SL-E	PrO	PoO	IL	CPCS	Source
*Calamaria andersoni*																
SYS r001699*	M	Tongbiguan, Yingjiang	319	32	9.1	9/9	13-13-13	171	23	4/4	2-3/2-3	1/1	1/1	5/5	at the foremost ends	Yang and Zheng (2018)
ANU ZR25022	M	Mangshi, Dehong	258	24	8.5	9/8	13-13-13	170	20	4/4	2-3/2-3	1/1	1/1	5/5	at the foremost ends	This study
ANU ZR24017	M	Mangdong, Lianghe, Dehong	269	26	8.8	9/9	13-13-13	168	22	4/4	2-3/2-3	1/1	1/1	5/5	at the foremost ends	This study
QHU 2025016	M	Mangdong, Lianghe, Dehong	240	21	8.0	9/9	13-13-13	164	20	4/4	2-3/2-3	1/1	1/1	5/5	at the foremost ends	This study
QHU 2025017	M	Mangdong, Lianghe, Dehong	241	23	8.7	9/9	13-13-13	166	20	4/4	2-3/2-3	1/1	1/1	5/5	at the foremost ends	This study
QHU 2025018	M	Mangdong, Lianghe, Dehong	254	25	9.0	9/9	13-13-13	172	21	4/4	2-3/2-3	1/1	1/1	5/5	at the foremost ends	This study
HS R21036	F	Mangdong, Lianghe, Dehong	294	18	5.8	9/9	13-13-13	186	14	4/4	2-3/2-3	1/1	1/1	5/5	at the foremost ends	This study
*Calamaria yunnanensis*																
ZISP 17073*	M	Jingdong	225	20	8.1	n/a	13-13-13	173	20	4/4	2-3/2-3	0/0	1/1	5/5	n/a	Lee (2021)
ROM 41547	M	Simao	226	20	8.1	8	13-13-13	167	19	4/4	2-3/2-3	0/0	1/1	5/5	n/a	Lee (2021)
KIZ 056009	M	Jingdong	280+	20	n/a	n/a	13-13-13	184+	20	4/4	2-3/2-3	0/0	1/1	5/5	n/a	Lee (2021)
KIZ 056010	M	Jingdong	280	16	5.4	9	13-13-13	179	15	4/4	2-3/2-3	0/0	1/1	5/5	n/a	Lee (2021)
KIZ 056011	SM	Jingdong	136	8	5.6	9	13-13-13	181	16	4/4	2-3/2-3	0/0	1/1	5/5	n/a	Lee (2021)
QHU R2024054	M	Mt. Wanzhang, Simao	263	24	8.4	9/9	13-13-13	167	21	4/4	2-3/2-3	0/0	1/1	5/5	about anterior 1/2	This study
QHU R2024055	F	Mengsong, Mengla, Xishuangbanna	411	24	5.5	9/9	13-13-13	189	16	4/4	2-3/2-3	0/0	1/1	5/5	about anterior 1/2	This study
KIZ 054176	F	Jingdong	490	26	5.0	9	13-13-13	199	19	4/4	2-3/2-3	0/0	1/1	5/5	n/a	Lee (2021)
