# Supplementary material for: First description of the female of Calamaria andersoni Yang & Zheng, 2018 (Squamata, Calamariidae), with an expanded diagnosis
Source: Biodivers Data J. 2025 Dec 15;13:e165597. doi: 10.3897/BDJ.13.e165597 (PMC12723395; doi:10.3897/BDJ.13.e165597)
Supplement: Supplementary material 1 — Tables S1–S4 [file bdj-13-e165597-s001.doc]

**First description of the female specimen of Clamaria andersoni Yang & Zheng, 2018 (Squamata: Calamariidae), with an expanded diagnosis**

**Tierui Zhang^1^, Yuhao Xu^2^, Tan Van Nguyen^3,4,*^**

**Nikolay A. Poyarkov^5^, Lifang Peng^2^, Jundong Deng^2^, Xinge Wang^1^, Song Huang^1,*^**

*1 The Anhui Provincial Key Laboratory of Biodiversity Conservation and Ecological Security in the Yangtze River Basin*, *College of Life Sciences*, *Anhui Normal University*, *Wuhu 241000*, *Anhui*, *China*

*2 State Key Laboratory of Plateau Ecology and Agriculture*, *Qinghai University*, *Xining 810016*, *China*

*3 Institute for Research and Training in Medicine*, *Biology and Pharmacy*, *Duy Tan University*, *Da Nang*, *550000*, *Vietnam*

*4 College of Medicine and Pharmacy*, *Duy Tan University*, *120 Hoang Minh Thao*, *Lien Chieu*, *Da Nang*, *550000*, *Vietnam*

*5 Department of Vertebrate Zoology*, *Lomonosov Moscow State University*, *Leninskiye Gory*, *GSP–1*, *Moscow 119991*, *Russia*

Corresponding authors: Tan Van Nguyen ([tan.sifasv@gmail.com](mailto:tan.sifasv@gmail.com)); Song Huang ([snakeman@ahnu.edu.cn](mailto:snakeman@ahnu.edu.cn)).

**Table S1**. DNA sequences, voucher specimens, and GenBank accession numbers of the genus *Calamaria* and outgroup taxa used in this study.

| **Species** | **Specimen voucher** | **Locality** | **Cyt *b*** | **Soures** |
| --- | --- | --- | --- | --- |
| *C*. *alcalai* | PNM 9873 | Sitio Palbong, Barangay Batong Buhay, Sablayan, Mindoro, Philippines | MT819383 | Weinell et al. 2021 |
| *C*. *andersoni* | SYS r001699 | Yingjiang, Yunnan, China | MH445955 | Yang & Zheng 2018 |
| *C*. *andersoni* | HS R20101 | Dehong, Yunnan, China | OQ354844 | Cai et al. 2023 |
| *C*. *andersoni* | HS R20181 | Tengchong, Yunnan, China | OQ354845 | Cai et al. 2023 |
| *C*. *andersoni* | ANU ZR25022 | Mangshi, Dehong, Yunnan, China | PV745123 | This study |
| *C*. *andersoni* | ANU ZR24017 | Mangdong, Lianghe, Dehong, Yunnan, China | PV915791 | This study |
| *C*. *andersoni* | **HS R21036** | Mangdong, Lianghe, Dehong, Yunnan, China | PV915788 | This study |
| *C*. *andersoni* | QHU R2025016 | Mangdong, Lianghe, Dehong, Yunnan, China | PV915789 | This study |
| *C*. *andersoni* | QHU R2025017 | Mangdong, Lianghe, Dehong, Yunnan, China | PV915790 | This study |
| *C*. *andersoni* | QHU R2025018 | Mangdong, Lianghe, Dehong, Yunnan, China | PV915792 | This study |
| *C*. *arcana* | KFBG 14611 | Mt. Dadongshan, Guangdong, China | ON482335 | Yeung et al. 2022 |
| *C*. *arcana* | HS 17082 | Mt. Dawu, Guangdong, China | OQ354835 | Cai et al. 2023 |
| *C*. *arcana* | GP 9975 | Yongxing, Hunan, China | OP980549 | Cai et al. 2023 |
| *C*. *arcana* | DL R199 | Mt. Wuyi, Fujian, China | OQ354834 | Cai et al. 2023 |
| *C*. *berezowskii* | GXNU DLR194 | Mt. Gongga, Sichuan, China | PP747047 | Liang et al. 2024 |
| *C*. *berezowskii* | GXNU DLR195 | Mt. Gongga, Sichuan, China | PP747048 | Liang et al. 2024 |
| *C*. *berezowskii* | GXNU 20221215002 | Mt. Gongga, Sichuan, China | PP747049 | Liang et al. 2024 |
| *C*. *gervaisii* | KU 324661 | Puguis, La Trinidad, Benguet, Luzon, Philippines | MT819384 | Weinell et al. 2021 |
| *C*. *gervaisii* | KU 334485 | Narvacan, Ilocos Sur, Luzon, Philippines | MT819385 | Weinell et al. 2021 |
| *C*. *jinggangensis* | DL 20200725 | Mt. Jinggangshan, Jiangxi, China | OQ354830 | Cai et al. 2023 |
| *C*. *jinggangensis* | DL 20200625-2 | Mt. Jinggangshan, Jiangxi, China | OQ354831 | Cai et al. 2023 |
| *C*. *jinggangensis* | DL 20200625-3 | Mt. Jinggangshan, Jiangxi, China | OQ354832 | Cai et al. 2023 |
| *C*. *jinggangensis* | DL 20200625-4 | Mt. Jinggangshan, Jiangxi, China | OQ354833 | Cai et al. 2023 |
| *C*. *lumbricoidea* | KU 315159 | Pasonanca NP, Zamboanga, Philippines | MT819388 | Weinell et al. 2021 |
| *C*. *lumbricoidea* | KU 334479 | Mt. Lumot, Gingoog, Misamis, Philippines | MT819389 | Weinell et al. 2021 |
| *C*. cf. *lumbricoidea* | USMHC 1560 | Air Itam Dam, Penang, Malaysia | MN338526 | Quah et al. 2019 |
| *C*. *muelleri* | TNHC 58955 | Gowa, South Sulawesi, Indonesia | MT819390 | Weinell et al. 2021 |
| *C*. *muelleri* | RMB 1283 | Gowa, South Sulawesi, Indonesia | MT819391 | Weinell et al. 2021 |
| *C*. *nebulosa* | FMNH 258666 | Phongsaly, Laos | MN338524 | Quah et al. 2019 |
| *C*. *palavanensi* | KU 309445 | Barangay Irawan, Puerto Princessa, Palawan, Philippines | MT819386 | Weinell et al. 2021 |
| *C*. *palavanensi* | KU 311411 | Mt. Mantalingahan, Rizal, Palawan, Philippines | MT819387 | Weinell et al. 2021 |
| *C*. *pavimentata* | KFBG 14507 | Ningming, Guangxi, China | MH445957 | Yang & Zheng 2018 |
| *C*. cf. *pavimentata* | MZMU 1269 | Mizoram, India | MN788502 | Kundu et al.  unpublished data |
| *C*. *schlegeli* | LSUHC 10278 | Bukit Larut, Perak, Malaysia | MN338525 | Quah et al. 2019 |
| *C*. *septentrionalis* | KFBG 14506 | Hainan, China | MH445956 | Yang & Zheng 2018 |
| *C*. *septentrionalis* | HS 12055 (CHS 118) | Huangshan, Anhui, China | MK201274 | Li et al. 2020 |
| *C*. *septentrionalis* | RE 30 (CHS 302) | Mangshan, Hunan, China | MK201384 | Li et al. 2020 |
| *C*. *septentrionalis* | HS 11145 | Mt. Nanling, Guangdong, China | OQ354840 | Cai et al. 2023 |
| *C*. *septentrionalis* | DL 2021610-1 | Huangsha, Guangxi, China | OQ354838 | Cai et al. 2023 |
| *C*. cf. *septentrionalis* | ROM 35605 | Phia Oac-Phia Den NP, Cao Bang, Vietnam | AF471081 | Lawson et al. 2005 |
| *C*. cf. *septentrionalis* | ROM 35597 | Phia Oac-Phia Den NP, Cao Bang, Vietnam | KX694890 | Alencar et al. 2016 |
| *C*. *yunnanensis* | ROM 41547 | Simao, Yunnan, China | KX694891 | Zaher et al. 2009 |
| *C*. *yunnanensis* | YPx 503 | Yunnan, China | JQ598922 | Grazziotin et al. 2012 |
| *C*. *yunnanensis* | QHU R2024054 | Mt. Wanzhangshan, Simao, Yunnan, China | PV755783 | This study |
| **Outgroup** |  |  |  |  |
| *Orientocoluber spinalis* | MVZ 211019 | Ningxia, China | AY486924 | Nagy et al. 2004 |
| *Elaphe quatuorlineata* | LSUMZ 40626 | Hungary | AY486931 | Nagy et al. 2004 |
| *Lycodon rufozonatus* | LSUMZ 44977 | China | AF471063 | Lawson et al 2005 |

**Table S2.** Museums, private collections and biorepository abbreviations mentioned in this study.

| ANU: Anhui Normal University, Wuhu, China |
| --- |
| DL: Ding Lee’s private collection, Chengdu Institute of Biology, Sichuan, China |
| FMNH: Field Museum of Natural History, Chicago, USA |
| FTB: Frank T. Burbrink field tag series |
| GP: Guo Peng private collection, Yibin University, Yibin, China |
| GXNU: Guangxi Normal University, Guangxi, China |
| HS: Song Huang field tag series, Anhui Normal University, Anhui, China |
| KFBG: Herpetological Collection of Kadoorie Farm and Botanic Garden, Hong Kong, China |
| KIZ: Kunming Institute of Zoology, Yunnan, China |
| KU: Museum of Natural History, University of Kansas, Lawrence, USA |
| LSUHC: La Sierra University Herpetological Collections, Riverside, USA |
| LSUMZ: Louisiana State University Museum of Natural Science, Louisiana, USA |
| MVZ: Museum of Vertebrate Zoology, University of California at Berkeley, Berkeley, California, USA |
| MZMU: Departmental Museum of Zoology, Mizoram University, Mizoram, India |
| QHU: Qinghai University, Xining, China |
| RMB: Rafe M. Brown field tag (specimen deposited in Museum Zoologicum Bogoriense, Indonesia) |
| ROM: Royal Ontario Museum, Toronto, Ontario, Canada |
| SYS: Sun Yat-Sen University, Guangzhou, Guangdong, China |
| TNHC: Texas Natural History Collections, University of Texas at Austin, Texas, USA |
| USMHC: Universiti Sains Malaysia Herpetological Collection, Penang, Malaysia |

**Table S3.** Uncorrected *p*-distances (%) among *Calamaria* species based on 1,105 base pairs from the mitochondrial genes Cyt *b*. The serial numbers in Table S3 are consistent with those in Table S1.

| **No.** | **Cyt *b*** | **Specimen** | **1** | **2** | **3** | **4** | **5** | **6** | **7** | **8** | **9** | **10** | **11** | **12** |
| --- | --- | --- | --- | --- | --- | --- | --- | --- | --- | --- | --- | --- | --- | --- |
| **1** | PV915788 | *Calamaria andersoni* (**HS R21036**) | | |  |  |  |  |  |  |  |  |  |  |
| **2** | PV745123 | *Calamaria andersoni* (ANU ZR25022) | 0.2 |  |  |  |  |  |  |  |  |  |  |  |
| **3** | PV915791 | *Calamaria andersoni* (ANU ZR24017) | 0.3 | 0.2 |  |  |  |  |  |  |  |  |  |  |
| **4** | PV915789 | *Calamaria andersoni* (QHU R2025016) | 0.3 | 0.1 | 0.2 |  |  |  |  |  |  |  |  |  |
| **5** | PV915790 | *Calamaria andersoni* (QHU R2025017) | 0.4 | 0.2 | 0.1 | 0.1 |  |  |  |  |  |  |  |  |
| **6** | PV915792 | *Calamaria andersoni* (QHU R2025018) | 0.3 | 0.1 | 0.3 | 0.3 | 0.4 |  |  |  |  |  |  |  |
| **7** | MH445955 | *Calamaria andersoni* (SYS r001699) | 2.5 | 2.5 | 2.5 | 2.4 | 2.5 | 2.4 |  |  |  |  |  |  |
| **8** | OQ354844 | *Calamaria andersoni* (HSR 20101) | 0.1 | 0.1 | 0.1 | 0.1 | 0.2 | 0.1 | 2.4 |  |  |  |  |  |
| **9** | OQ354845 | *Calamaria andersoni* (HSR 20181) | 0.0 | 0.2 | 0.2 | 0.1 | 0.2 | 0.1 | 2.5 | 0.1 |  |  |  |  |
| **10** | MT819383 | *Calamaria alcalai* | 21.8 | 21.8 | 21.8 | 21.7 | 21.8 | 21.7 | 21.8 | 21.7 | 21.8 |  |  |  |
| **11** | ON482335 | *Calamaria arcana* | 16.4 | 16.7 | 16.7 | 16.6 | 16.7 | 16.6 | 16.0 | 16.6 | 16.4 | 19.6 |  |  |
| **12** | PP747049 | *Calamaria berezowskii* | 16.1 | 16.3 | 16.1 | 16.2 | 16.1 | 16.2 | 16.2 | 16.2 | 16.1 | 21.1 | 15.3 |  |
| **13** | MT819384 | *Calamaria gervaisii* | 20.7 | 20.7 | 20.7 | 20.6 | 20.7 | 20.6 | 20.0 | 20.6 | 20.7 | 17.3 | 19.7 | 20.7 |
| **14** | OQ354830 | *Calamaria jinggangensis* | 16.4 | 16.6 | 16.6 | 16.5 | 16.6 | 16.5 | 16.3 | 16.5 | 16.4 | 20.5 | 5.7 | 14.6 |
| **15** | MN338526 | *Calamaria* cf. *lumbricoidea* | 21.3 | 21.3 | 21.1 | 21.2 | 21.1 | 21.2 | 21.2 | 21.2 | 21.3 | 16.5 | 20.4 | 19.9 |
| **16** | MT819389 | *Calamaria lumbricoidea* | 18.8 | 18.8 | 18.8 | 18.7 | 18.8 | 18.7 | 19.7 | 18.7 | 18.8 | 14.9 | 19.2 | 19.8 |
| **17** | MT819391 | *Calamaria muelleri* | 21.2 | 21.5 | 21.2 | 21.2 | 21.2 | 21.2 | 20.2 | 21.2 | 21.2 | 14.5 | 17.2 | 19.9 |
| **18** | MN338524 | *Calamaria nebulosa* | 17.8 | 17.8 | 17.6 | 17.7 | 17.6 | 17.7 | 17.2 | 17.7 | 17.8 | 21.4 | 16.4 | 16.6 |
| **19** | MT819386 | *Calamaria palavanensis* | 20.4 | 20.7 | 20.4 | 20.4 | 20.4 | 20.4 | 20.7 | 20.4 | 20.4 | 14.9 | 21.0 | 23.4 |
| **20** | MH445957 | *Calamaria pavimentata* | 18.8 | 18.8 | 18.6 | 18.7 | 18.6 | 18.7 | 18.6 | 18.7 | 18.8 | 21.8 | 17.0 | 15.8 |
| **21** | MN788502 | *Calamaria* cf. *pavimentata* | 13.1 | 13.3 | 13.1 | 13.1 | 13.1 | 13.1 | 13.3 | 13.1 | 13.1 | 22.0 | 10.0 | 16.2 |
| **22** | MN338525 | *Calamaria schlegeli* | 20.3 | 20.3 | 20.3 | 20.2 | 20.3 | 20.2 | 20.6 | 20.2 | 20.3 | 14.7 | 19.9 | 21.1 |
| **23** | MK201434 | *Calamaria septentrionalis* | 13.8 | 14.2 | 13.8 | 14.0 | 13.8 | 14.0 | 14.9 | 14.0 | 13.8 | 21.1 | 11.0 | 15.2 |
| **24** | KX694890 | *Calamaria* cf. *septentrionalis* | 14.2 | 14.5 | 14.5 | 14.3 | 14.5 | 14.3 | 14.8 | 14.3 | 14.2 | 21.7 | 10.6 | 14.2 |
| **25** | KX694891 | *Calamaria yunnanensis* | 9.8 | 10.1 | 10.1 | 9.9 | 10.1 | 9.9 | 9.8 | 9.9 | 9.8 | 21.7 | 16.3 | 17.1 |

**Table S3.** Continued.

| No. | Cyt b | Species | 13 | 14 | 15 | 16 | 17 | 18 | 19 | 20 | 21 | 22 | 23 | 24 |
| --- | --- | --- | --- | --- | --- | --- | --- | --- | --- | --- | --- | --- | --- | --- |
| 14 | OQ354830 | *Calamaria jinggangensis* | 19.2 |  |  |  |  |  |  |  |  |  |  |  |
| 15 | MN338526 | *Calamaria* cf. *lumbricoidea* | 18.6 | 20.9 |  |  |  |  |  |  |  |  |  |  |
| 16 | MT819389 | *Calamaria lumbricoidea* | 17.3 | 18.7 | 13.3 |  |  |  |  |  |  |  |  |  |
| 17 | MT819391 | *Calamaria muelleri* | 15.1 | 15.9 | 15.1 | 12.5 |  |  |  |  |  |  |  |  |
| 18 | MN338524 | *Calamaria nebulosa* | 21.0 | 16.5 | 20.0 | 18.4 | 18.0 |  |  |  |  |  |  |  |
| 19 | MT819386 | *Calamaria palavanensis* | 15.8 | 18.2 | 16.4 | 16.5 | 15.5 | 19.1 |  |  |  |  |  |  |
| 20 | MH445957 | *Calamaria pavimentata* | 22.0 | 15.7 | 21.5 | 20.4 | 21.8 | 17.7 | 22.2 |  |  |  |  |  |
| 21 | MN788502 | *Calamaria* cf. *pavimentata* | 18.1 | 9.4 | 18.8 | 18.3 | 18.8 | 14.6 | 19.3 | 11.6 |  |  |  |  |
| 22 | MN338525 | *Calamaria schlegeli* | 17.8 | 19.0 | 16.7 | 14.4 | 15.6 | 18.2 | 16.7 | 19.7 | 18.1 |  |  |  |
| 23 | MK201434 | *Calamaria septentrionalis* | 21.5 | 9.7 | 21.0 | 20.5 | 20.3 | 18.1 | 19.5 | 15.0 | 7.0 | 20.2 |  |  |
| 24 | KX694890 | *Calamaria* cf. *septentrionalis* | 21.2 | 10.4 | 20.1 | 20.1 | 20.2 | 17.1 | 20.1 | 16.6 | 7.5 | 19.8 | 4.4 |  |
| 25 | KX694891 | *Calamaria yunnanensis* | 22.0 | 14.9 | 21.2 | 20.6 | 22.3 | 16.9 | 22.2 | 17.7 | 16.4 | 20.6 | 14.9 | 14.2 |

**Table S4.** Overview measurements of the examined specimens of Calamaria andersoni and C. yunnanensis from Yunnan, China. **Notes:** n/a = not available; * = Holotype; M = male; F = female; SM = subadult male; HL = head length; HW = head width; ED = eye diameter; Eye-MouthD = eye-mouth distance; NarEye = naris–eye distance; IOD = interorbital distance.

| **Specmen number** | **Sex** | **Locality** | **HL** | **HW** | **ED** | **Eye–MouthD** | **NarEye** | **IOD** | **Source** |
| --- | --- | --- | --- | --- | --- | --- | --- | --- | --- |
| ***Calamaria andersoni*** | | | | | | | | | |
| SYS r001699* | M | Tongbiguan, Yingjiang | 6.5 | 5.2 | n/a | n/a | n/a | n/a | Yang & Zheng 2018 |
| ANU ZR25022 | M | Mangshi, Dehong | 8.0 | 4.5 | 1.0 | 1.0 | 1.5 | 3.1 | This study |
| ANU ZR24017 | M | Mangdong, Lianghe, Dehong | 8.1 | 5.0 | 1.0 | 0.9 | 1.6 | 3.3 | This study |
| QHU 2025016 | M | Mangdong, Lianghe, Dehong | 7.5 | 4.8 | 0.9 | 0.9 | 1.4 | 3.3 | This study |
| QHU 2025017 | M | Mangdong, Lianghe, Dehong | 7.7 | 4.8 | 0.9 | 0.8 | 1.6 | 3.3 | This study |
| QHU 2025018 | M | Mangdong, Lianghe, Dehong | 8.0 | 5.5 | 1.1 | 0.9 | 1.5 | 3.5 | This study |
| **HS R21036** | F | Mangdong, Lianghe, Dehong | 7.8 | 4.2 | 0.9 | 0.9 | 1.6 | 3.2 | This study |
| ***Calamaria yunnanensis*** | | | | | | | | | |
| ZISP 17073* | M | Jingdong | n/a | n/a | n/a | n/a | n/a | n/a | Lee 2021 |
| ROM 41547 | M | Simao | 7.4 | 4.0 | 1.0 | n/a | 1.5 | 3.1 | Lee 2021 |
| KIZ 056009 | M | Jingdong | n/a | n/a | n/a | n/a | n/a | n/a | Lee 2021 |
| KIZ 056010 | M | Jingdong | 7.6 | 4.9 | 0.6 | n/a | 1.2 | 2.1 | Lee 2021 |
| KIZ 056011 | SM | Jingdong | 4.8 | 3.3 | 0.6 | n/a | 1.3 | 2.0 | Lee 2021 |
| QHU R2024054 | M | Mt. Wanzhang, Simao | 8.5 | 4.5 | 0.9 | 0.9 | 1.5 | 2.7 | This study |
| QHU R2024055 | F | Mengsong, Mengla, Xishuangbanna | n/a | 6.1 | 1.2 | 1.3 | n/a | 3.8 | This study |
| KIZ 054176 | F | Jingdong | 9.8 | 6.8 | 0.8 | n/a | 2.5 | 4.0 | Lee 2021 |

**Reference**

Alencar LRV, Quental TB, Grazziotin FG, Alfaro ML, Martins M, Venzon M, Zaher H (2016) Diversification in vipers: Phylogenetic relationships, time of divergence and shifts in speciation rates. Molecular Phylogenetics and Evolution 105: 50–62. https://doi.org/10.1016/j.ympev.2016.07.029

Cai B, Jiang JP, Wu YY, Huang S, Fei DB, Ding L (2023) A new species of *Calamaria* (Reptilia: Serpentes: Colubridae) from Western Jiangxi Province, China. Russian Journal of Herpetology 30(2): 101–111. <https://doi.org/10.30906/1026-2296-2023-30-2-101-111>

Grazziotin FG, Zaher H, Murphy RW, Scrocchi G, Benavides MA, Zhang YP, Bonatto SL (2012) Molecular phylogeny of the new world Dipsadidae (Serpentes: Colubroidea): a reappraisal. Cladistics 28(5): 437–459. https://doi.org/10.1111/j.1096-0031.2012.00393.x

Lawson R, Slowinski JB, Crother BI, Burbrink FT (2005) Phylogeny of the Colubroidea (Serpentes): New evidence from mitochondrial and nuclear genes. Molecular Phylogenetics and Evolution 37(2): 581–601. <https://doi.org/10.1016/j.ympev.2005.07.016>

Lee JL (2021) Description of a new species of Southeast Asian reed snake from northern Laos (Squamata: Colubridae: Genus Calamaria F. Boie, 1827) with a revised diagnosis of Calamaria yunnanensis Chernov, 1962. Journal of Natural History 55(9–10): 531–560. https://doi.org/10.1080/00222933.2021.1909165

Li JN, Liang D, Wang YY, Guo P, Huang S, Zhang P (2020) A large-scale systematic framework of Chinese snakes based on a unified multilocus marker system. Molecular Phylogenetics and Evolution 148 https://doi.org/10.1016/j.ympev.2020.106807

Liang YT, Huang ZD, Ding L, Vogel G, Ananjeva NB, Orlov NL, Shi SC, Wu ZJ, Chen ZN (2024) Revalidated after having been described more than a century ago: *Calamaria berezowskii* Günther, 1896 (Squamata, Colubridae) from Sichuan, Southwestern China. Zoosystematics and Evolution 100 (3): 897‑911. <https://doi.org/10.3897/zse.100.125798>

Nagy ZT, Lawson R, Joger U, Wink M (2004) Molecular systematics of racers, whipsnakes and relatives (Reptilia: Colubridae) using mitochondrial and nuclear markers. Journal of Zoological Systematics and Evolutionary Research 42(3): 223–233. https://doi.org/10.1111/j.1439-0469.2004.00249.x

Quah ESH, Anuar S, Grismer LL, Wood PL, Mohd Nor SA (2019) Systematics and natural history of mountain reed snakes (genus *Macrocalamus*; Calamariinae). Zoological Journal of the Linnean Society 188 (4): 1236‑1276. https://doi.org/10.1093/zoolinnean/zlz092

Weinell J, Leviton AE, Brown RM (2021) A new species of reed snake, genus *Calamaria* (Colubridae: Calamariinae), from Mindoro Island, Philippines. Philippine Journal of Systematic Biology 14 (2). <https://doi.org/10.26757/pjsb2020b14006>

Yang JH, Zheng X (2018) A new species of the genus *Calamaria*(Squamata: Colubridae) from Yunnan Province, China. Copeia 106(3): 485–491. https://doi.org/10.1643/CH-17-663

Yeung HY, Lau MWN, Yang JH (2022) A new species of *Calamaria* (Squamata: Colubridae) from Guangdong Province, southern China. Vertebrate Zoology 72: 433–444. <https://doi.org/10.3897/vz.72.e84516>

Zaher H, Grazziotin FG, Cadle JE, Murphy RW, Moura-Leite JCD, Bonatto SL (2009) Molecular phylogeny of advanced snakes (Serpentes, Caenophidia) with an emphasis on South American Xenodontines: A revised classification and descriptions of new taxa. Papéis Avulsos de Zoologia 49(11): 115–153. https://doi.org/10.1590/S0031-10492009001100001
